# Supplementary material for: Patterns of Intron Gain and Loss in Fungi
Source: PLoS Biol. 2004 Nov 30;2(12):e422. doi: 10.1371/journal.pbio.0020422 (PMC532390; doi:10.1371/journal.pbio.0020422)
Supplement: Table S1 — Also available at http://genes.mit.edu/NielsenEtAl/. (4.3 MB ZIP). [file pbio.0020422.st001.zip › NielsenEtAl/html/70.html]

AN3363.1.NCU00927.1.MG01175.1.FG00697.1


```
 CLUSTAL W (1.82) Multiple Sequence Alignments - Introns Inserted


Sequence 1: MG01175.1	1191 aa
Sequence 2: FG00697.1	1034 aa
Sequence 3: NCU00927.1	1204 aa
Sequence 4: AN3363.1	1184 aa
Alignment Length: 1248 aa
Number Identitical Residues: 369 aa
Alignment Score (without introns) 20436


MG01175.1 	MSSLREPSRRLPRASTASSAGASTTSRTGAIGRASVRPGVRAPSTSTRNVRLNHHSAIAS
NCU00927.1	-------MERKKPSSMSRPPSVAGTRSNSSMRPPPTRN--TASSTGRQSALRNASAPGGS
FG00697.1 	------------------------------------------------------------
AN3363.1  	-------------MAGPQRATSGLPTRRTTTRQPTRRAGSAIPERQTSTASPAVSTKTAA
          	              : .  .  . .    :   ..   .   ..    .      :  .:

MG01175.1 	RIDRSGAASPVESVSSMATTMTGGMKRKERDYEAAEVGLGEKTNINVVVRCRGRNDREVR
NCU00927.1	RAERSGAASPADS----QVSATAGAKRKERDFDPEDEGT---TNINVVVRCRGRNDREVK
FG00697.1 	------------------------------------------------------------
AN3363.1  	ISRTRTLKSPGEP-------ASVLAKRKERDIER---EINEDTSIHVVVRCRGRNEREVK
          	        :. ..        :   . .. . .      ...:.      . . .. .  

MG01175.1 	ENSAVVVSTEGVKGNVVELSMGPNALSNKSYNFDRVFSSAADQSMIFDDVVKPILDE0ML
NCU00927.1	ENSAVVVKTEGLKGRIVELSMGPNAVSNKTYTFDRVFSQAADQAMVFDEVVKPILEE0ML
FG00697.1 	---------------------------------------------------------~ML
AN3363.1  	ENSGVVLQTEGVKGKTVELSMGPNAVSNKTYTFDKVFSAAADQITVYEDVVLPIVTE0ML
          	..:.   .:.. ..   . : ...: :..: . .   : ::..    ..   .   . **

MG01175.1 	SGFNCTIFAYGQTGTGKTYTMTGDMNETMGMLSDAAGIIPRVLQTLFNKLEIEESDNCVR
NCU00927.1	AGYNCTIFAYGQTGTGKTYTMSGDMNQTFGMLPDTAGIIPRVLHALFNKLEVDDKEHCVR
FG00697.1 	SGYNCTIFAYGQTGTGKTYTMSGDMTETMGMLSDDAGIIPRVLQTLFTKLELDNAESTIK
AN3363.1  	AGYNCTIFAYGQTGTGKTYTMSGDMTDTLGILSDNAGIIPRVLYSLFAKLA--DTESTVK
          	:*:******************:***.:*:*:*.* ******** :** **   : :  ::

MG01175.1 	CSFIELYNEELRDLLSADDGAKLRIYEDNAKK---STVVQGMEERHIVTAAEGIKRLQEG
NCU00927.1	CSFIELYNEELRDLLSAEDNTKLKIFDDNSKKGHATTIVQGMEERHILSAADGLRWLQEG
FG00697.1 	CSFIELYNEELRDLLASDEGTKLKIYDDTSRRGHASTIVQGMEEKHIKDAAEGVKVLQEG
AN3363.1  	CSFIELYNEELRDLLSAEENPKLKIYDNEQKKGHMSTLVQGMEETYIDSATAGIKLLQQG
          	***************::::..**:*:::  ::.  :*:****** :*  *: *:: **:*

MG01175.1 	SLRRQVAATKCNDLSSRSHTIFTITVYVKRPGENG-DDYVSAGKLNLVDLAGSENIQRSG
NCU00927.1	SVRRQVAATKCNDLSSRSHTVFTITVHVKQQTENG-EDYLMGGKLNLVDLAGSENIQRSG
FG00697.1 	SLKRQVAATKCNDLSSRSHTVFTITTYVRKPNEHGVEALVSAGKLNLVDLAGSENIQRSG
AN3363.1  	SHKRQVAATKCNDLSSRSHTVFTITVNIKRTTESG-EEYVCPGKLNLVDLAGSENIGRSG
          	* :*****************:****. :::  * * :  :  ************** ***

MG01175.1 	AENKRAAEAGLINKSLLTLGRVINSLVDQGTHIPYR~ESKLTRLLQDSLGGRTKTCIIAT
NCU00927.1	AENKRAAEAGLINKSLLTLGRVINALVDRSPHIPYR~ESKLTRLLQDSLGGRTKTCIIAT
FG00697.1 	AENKRATEAGLINKSLLTLGRVINALVDKGSHIPYR~ESKLTRLLQDSLGGRTKTCIIAT
AN3363.1  	AENKRATEAGLINKSLLTLGRVINALVDKSQHIPYR2ESKLTRLLQDSLGGRTKTCIIAT
          	******:*****************:***:. ***** ***********************

MG01175.1 	ISPAKSNLEETISTLDYAFRAKDIRNKPQIRTLMSKKTLLREFTAEIEKLRGELVTTRQR
NCU00927.1	ISPARSNMEETISTLDYAFRAKNIRNKPQINALLNKKTLLREFATEIEKLKSELIATRQR
FG00697.1 	ISPAKINLEETISTLEYAFRAKNIKNKPQMNPMIEKKTLLKDFTMEIERLKSELIATRQR
AN3363.1  	ISPARSNLEETISTLDYAFRAKNIRNKPQINSTMPKMTLLREFTAEIEKLKAELIATRHR
          	****: *:*******:******:*:****:.. : * ***::*: ***:*:.**::**:*

MG01175.1 	NGVYLSNEAYEEITVQNESRRILTEEQAAKIGTIEANLRGKVQELFDLTSSFMGLKKEHE
NCU00927.1	NGVYLSNDAYEELTVQNESRRILTEEQAARIETLETNLRNKLQELYTMTSTFMGLRKEHE
FG00697.1 	NGVYLSNESYEEMTAQSESRRIVNEEQSAKLDTLEKNLRNKVQELFSLQSTFLGLKKDHE
AN3363.1  	NGVYMSVESYEEMKMENESRRIISEEQRAKIESMESSLRHKVQELLTLTSKFNDLKKDND
          	****:* ::***:. :.*****:.*** *:: ::* .** *:***  : *.* .*:*:::

MG01175.1 	GTRLQLDDTKELLDQTELVLSGTRKSLADETYLRKAHQETEEKLANIGSELIDTLGETVK
NCU00927.1	ATKAQLDETKDVLDQTELVLAATRKSLAEEAHLRRAHQTTEQKLAKVGDELISTVKRTVQ
FG00697.1 	GTRAQLDDTKEVLDQTEIVLSATRQSLSEETKIRKAHQKTEQKLTEVGGELIDKLHKTVS
AN3363.1  	DTLAALCSTNDVLQQTDIVLQNTRAQLEEEEMLRCAHEETEHQLQDVGKGLISTLGQTVE
          	 *   * .*:::*:**::**  ** .* :*  :* **: **.:* .:*  **..: .**.

MG01175.1 	HVSGLRAKNKRKSDLQSLNRSTWAMSQAQVADVTELVEDRISEFQKDQEDHISSVSERMQ
NCU00927.1	DVEGLHAKNRRRSDLHALNRSTWAMSQSQVTGITELVESRIAEFRKGQEEHISSVSERMQ
FG00697.1 	DVGGLHAKNRRKSDLQSINRNTWTTSQNQVADVTSMVERRIGEFQDEQQEHIASVGQRMG
AN3363.1  	DINSLQSKLDRKAELDATNAELWRASSTEVSDVTKRIDQRVEAFQTRHAKLLETTSVKVN
          	.: .*::*  *:::*.: * . *  *. :*:.:*. :: *:  *:  : . : :.. :: 

MG01175.1 	SFVKAELDKLSDTQNLLDQNLGKFEISKKELLDHKEQSKQEMDAVLEEIKVVRDHIKERV
NCU00927.1	SFVQEELDKLSTTQAFLDQNLEHFAESRTQLLEQKQKSKEEMDNVLEEIKVVRDTVKQRV
FG00697.1 	SFVDEELRKLSSTQAFLDEHLGTFADSKKELLESKQKSKDDMDGVLEEIKVVRDTVKERM
AN3363.1  	EFIATEISNIERTRSDLSEYNRSLDAACNNAKAETSSAHEDMNNVLEEIKDLREEVKSKV
          	.*:  *: ::. *:  *.:    :  : .:    ...::::*: ****** :*: :*.::

MG01175.1 	GESLEAIASAAERIAADVLSELGLFHN----------------Q~LHTSFSSLGKDFKSI
NCU00927.1	GESLQAIASAAERIAGDVLGELTTFHS----------------Q~LHASYSSLGKECKSI
FG00697.1 	GESLQSISHSAERIAADMMSEMTAFHEQVWYITFYLRAITNAPQ~LHNSYSALGKDFKSV
AN3363.1  	GEGLNGLSAAAARISEEVIGEFTQLHS----------------Q0LHTSFNNLGKDLKSI
          	**.*:.:: :* **: :::.*:  :*.                * ** *:. ***: **:

MG01175.1 	FEDLVKHLGAQRSESDRLRRQLEGASESLIQSNAATSARIQEIVAEERRQAAVQRKQLLA
NCU00927.1	FGDLLQTISAQKAEAERLRQELEGASQTIVESNATVSARIQEVLEEERRQAAEERQNLLL
FG00697.1 	FEELVKHITAQRAECDNLKRQLQSATNTIVLQNATISSRIQDALTEERRLAVDDRQKLMA
AN3363.1  	FETMATHLSEQKNEINRLRAELQSSNRQNIETTHKASAHLAQAIEEEHVAAEAEREILMS
          	*  :   :  *: * :.*: :*:.:..  :  .   *::: : : **:  *  :*: *: 

MG01175.1 	QITQLINSQGETQELRLADKAALLNQDLLSSTKAFEGSVTQYSDGMGTWVEKDGRVMADI
NCU00927.1	QIGSLINSQAEMQESRLAGKTALIRQAVMDSNATFEGSMSEYMDGMNAWNEKDSRLMEDV
FG00697.1 	QISTLINTQAETQESRMQATASEIQKTITSTSTNLEQAVDTYGEGMSSWDLKEGEMLEEV
AN3363.1  	QIKALVEESRQKQFARLRAKIDGVRTEISASGDMLEQATTQHDRQIDEWVFKSEQFAKDV
          	**  *:: . : *  *:  .   :.  :  :   :* :   :   :. *  *. ..  ::

MG01175.1 	NKSRENVKTKMKDDWA0AANKHSTCIQATTKSVHAETVRVVEEQKRDLDVQMQDLDSFVT
NCU00927.1	VKSRDVLKNKMKDDWA0N----------TTKSVHAETVRVVDAQLKDLDTQMQDLDQFVS
FG00697.1 	KKSRDQLKTKLKDDWT~AADDHSSSIQATAKSVHAETVRAVDEQIKDLDVQMEALDDFVT
AN3363.1  	NASKDEIRTKLQNDWE0AFDQRNSTIRKATESVHKETVRIVDVQVDDMGRQMEALDDFVA
          	  *:: ::.*:::**    .. .:    :::*** **** *: *  *:. **: **.**:

MG01175.1 	RARSQNSLHHSQHAASVHQLSQTVENSYAEISSHSKDAVERVRELGDEMEAEVDDIRAGL
NCU00927.1	RARSENSVHHEQHVVSMENLTSTVEKSFINIADHFGERFDRIRDLSGEMDNDAKILRESL
FG00697.1 	RARTENGHHHATHSQSVDALSNTVEESFGNISAHFKSTFDRVKNLGEEMEVDLGDLQDGL
AN3363.1  	KARSQNGRYRDAHIATLDTIATGVRDSYSSIEGRVENLTGRMNQFQQEATHHHATLEESI
          	:**::*. ::  *  ::. ::  *..*: .*  :  .   *:.::  *   .   :. .:

MG01175.1 	EPLDENVCQPLSNLRESIRDTLLQEYQPTGETPEKTQYTYPTELPRTAPHEMLLAGLNGG
NCU00927.1	EPLDESLRQPLAALREDIQSTKIREYEPTGDTPEKKRYEYPTDLPRTAGHETLIAGMQDK
FG00697.1 	EPLKDQLCQPLANLREDVTGAALQEYQPTGETPAKVQYHYPTDLPRTEDHDLIISSIDEV
AN3363.1  	APLSNDVRKPLTDLSSSFQNRSLEEYVATGVTPKKRKYDYISVLPSTESHEVLKSRLR-T
          	 **.:.: :**: * ... .  :.** .** ** * :* * : ** *  *: : : :   

MG01175.1 	ALDTPS---KSAPAPPVVFSDFEETTVKR-----ERSPSTSS------------------
NCU00927.1	PSTTPTTSPAKRATMTAVYSDIVVSPSRSPSKVPLSVPVSPRNNPFGTPGPATSTRNKSH
FG00697.1 	ITPTKPGE-STDKDATIVFADLDSSPHKM-----MTSPVRPATR------MSMVSASEHM
AN3363.1  	TKEMEVLPFNSDDQLSGPSSSPGGSPSKGFVYNDVEDEVGTHAP----------TVTNVN
          	          .    .   :.   :. :            .             : ..  

MG01175.1 	AVSSGLREVNPNVS--------VTTSAIQFNP-SASTMSLAPLSG--TDNNSSTMPVYRR
NCU00927.1	PMDPSLREVNPNLGGGTSVFGELSSSTMSMAPPSTSVIGFHANNTDHTEESATQVPLFRR
FG00697.1 	GMPSSLREVNPNVPG------SLTTGTANYNP-RSSIISMPPER---------TMPLFKR
AN3363.1  	PSNTGLREVDANVAAR-----PLVYSTGEKSTDQDGSPVVSPDSATEAEGMNGPPSKRRR
          	   ..****:.*: .       :  .: .  .   .   . .     :.      .  :*

MG01175.1 	STRSVSYNKDTLSKSTTGGKPPSVASGIDGRENVPPVGGFAASGRRKSPRLR
NCU00927.1	SNRRQS-PRVLAKKKITGHAGGIITEGLENMPVTTDASVGIAGNRRKTPRLG
FG00697.1 	PTR------VTRSTKKVGSRDPIIS---EGGENVLPTALEESLSRRKSPRIN
AN3363.1  	SNS-----VVADTKLPNKMLARRMAGMMEGRENVPPPGISNGRRLRGRPSP-
          	..          ..         ::   :.   .   .   .   *  *
```
